# Markers in blood and saliva for prediction of orthodontically induced inflammatory root resorption: a retrospective case controlled-study

**DOI:** 10.1186/s40510-017-0176-y

**Published:** 2017-09-18

**Authors:** Dilara Yashin, Oyku Dalci, Mohammed Almuzian, Jenkin Chiu, Rajiv Ahuja, Apurv Goel, M. Ali. Darendeliler

**Affiliations:** 1 Discipline of Orthodontics, Faculty of Dentistry, University of Sydney, Sydney, Australia; 2 Australian Proteome Analysis Facility (APAF), Sydney, Australia; 3 John Radcliffe Hospital, Oxford University Hospitals NHS Foundation Trust, Oxford, UK; 4 Eastman Dental Hospital, University College London Hospitals NHS Foundation Trust, London, UK

## Abstract

**Background:**

Hormonal and enzymatic factors may render certain individuals more susceptible to orthodontically induced inflammatory root resorption (OIIRR). The objectives of this study are (1) to identify biochemical key markers in blood and saliva that may be correlated to the trend of extensive OIIRR and (2) to utilise these markers to predict a susceptible patient-receiving orthodontic treatment.

**Methods:**

Nine patients (mean age 23 + 2.9 years) who had moderate to severe OIIRR that assessed via orthopantomograms and met the inclusion criteria were classified as the root resorption group (RRG). Blood chemistry was evaluated using the collection of fasting blood and unstimulated saliva samples. Multiplex enzyme-linked immunosorbent assay (ELISA) arrays were used to screen blood and saliva samples for human cytokines, chemokines and several key enzymes that may play a role in root resorption following orthodontic force application. Biochemical findings from 16 matching subjects were used as the control (CG) for comparative measurements.

**Results:**

Patients with moderate to severe OIIRR showed a significant increase in salivary cytokines including interleukin (IL) 7, IL-10, IL-12p70 and interferon-gamma (IFN-γ) level as well as a significant decrease in IL-4 level. Osteocalcin and procollagen type I N-terminal peptide (P1NP) appeared to be the only blood factors that showed a significant difference, more in the CG than the RRG.

**Conclusions:**

Saliva might be a more valuable way of measuring changes in cytokine expression than blood secondary to orthodontic treatment. Although the increased expression of pro-inflammatory and anti-inflammatory cytokines may be determinants in the development of moderate to severe OIIRR, cytokine expression may be affected by several potential inflammations in another part of the body. Future research could investigate the cause/effect relationship of different cytokines, in a larger group of patients and at different time intervals, using digital subtraction radiography techniques and microfluidic biosensors.

## Background

Orthodontically induced inflammatory root resorption (OIIRR) is not an uncommon iatrogenic consequence of orthodontic treatment [1]. The inflammatory component of OIIRR comes from force application inducing local biological changes that are essential for tooth movement and also the cause of root resorption process [2]. While in most patients the degree of root resorption is minor and barely noticeable, moderate to severe resorption can be exhibited in several teeth, especially in the aesthetic zone [3]. In their 1988 paper, Lavender and Malmgren quantified root resorption with an index in which grade 1 is an irregular root contour; grade 2 is apical root resorption of less than 2 mm; grade 3 is apical root resorption greater than 2 mm to one third of the original root length; and grade 4 is root resorption exceeding one third of the original root length [4].

There are limitations in finding clinical markers (factors) of root resorption during active orthodontic treatment. Although the anatomical risk factors have been investigated extensively [5–7], they account for only 20–30% of the expected variation in severity of OIIRR, indicating that they are not definitive predictors [5]. Some studies indicated that progress radiographs, taken at 6 to 12 months into treatment, could detect early OIIRR [7]. However, there is difficulty in using conventional radiography in detecting early levels of root demineralisation [8] because it takes approximately 30–60% of mineral content loss in order to visualise changes radiographically [8, 9]. Hence, conventional radiography should be used as an aid for comparison of treatment outcome as opposed to clinical markers for monitoring early changes in OIIRR.

Orthodontic movement is a micro-trauma to the periodontal ligament associated with a cascade of local periodontal inflammatory cycle [10]. Secondary to orthodontic movement, several local anti-resorptive and pro-resorptive cytokines have been observed [11–13]. Pro-resorptive cytokines such as interleukin (IL)-1 family (i.e. IL-1β), IL-6, IL-7, IL-8 and TNF-α. IL-1β which directly induce osteoclastogenesis and promote osteoclast function [14, 15]. Similarly, IL-6 acts synergistically with IL-1 and TNF-α on osteoclastogenesis and promotes osteoclast function [14, 15]. IL-7 works indirectly through the induction of TNF-α, an important augmenter of receptor activator of nuclear factor kappa-B ligand (RANKL) mediated osteoclastogenesis [16] while IL-8 enhances RANKL expression [17], both increase osteoclast generation and activates osteoclasts. On the other hand, the anti-resorptive cytokine such as IL-4 and interferon-gamma (IFN-γ) suppresses osteoclastogenesis and T-cell of RANKL-induced osteoclastogenesis, respectively [18, 19]. Granulocyte macrophage colony-stimulating factor (GM-CSF) is another anti-resorptive cytokine that inhibits bone resorption along with IL-4, IL-10, IL-13, IL-18 and IFN-γ [20].

There are several systemic biomarkers that could be related to OIIRR. For instance, the relationship between OIIRR and thyroxine hormone is still controversial; however it was found that the level of alkaline phosphatase rises significantly in patients with OIIRR [21]. Calcitonin has been proven to reduce orthodontic movement and thus may also affect root resorption [22]. On the other hand, an animal study showed that administration of osteocalcin could accelerate movement and subsequently might induce severe OIIRR [23]. Parathyroid hormone (PTH) acts synchronisingly with calcitonin to control the level of calcium in bone. Increased level of PTH is associated with skeletal calcium deficiency, faster tooth movement and may be increased OIIRR [24, 25]. There is also some evidence indicating that T-lymphocytes are contributor to the inflammatory response secondary to orthodontic force, OIIRR and bone remodelling [26]. Brezniak et al. stated that consumption of alcohol during orthodontic treatment is related to high risk of OIIRR due to vitamin D hydroxylation in the liver [27]. Similarly, Collins and Sinclair showed that the number of mononuclear osteoclasts boosted following administration of vitamin D in the periodontal ligament of rats; this was associated with faster tooth movement and may be related to higher OIIRR [28]. The elevated level of IgE in patients with an allergy has association, but not statistically significant, with an increased risk of OIIRR [29].

The primary aim of this pilot study is to identify key local, in saliva, and systemic, in blood, biological markers that are associated with OIIRR in patients with moderate to severe root resorption. Secondly, this paper aims to use these findings to develop a predictive model for identifying patients susceptible to extensive OIIRR in order to allow for primary prevention of this serious complication. The null hypothesis of this project stated that there are no correlations between biological markers found in blood or saliva and the severity of OIIRR.

## Methods

### Sample

This is a single centre study approved by the Ethics Review Committee of the Sydney Local Health District (Protocol No X11-0028). All participants who had had their treatment completed between 2010 and 2012 at the Orthodontic Department of Sydney Dental Hospital (SDH) had been screened. Thirty-five participants had been identified of having moderate to severe root resorption (Grades 3 and 4) of at least three or more teeth at the completion of their treatment. The diagnostic criteria by Levander and Malmgren were used for assessing the severity of OIIRR seen in digital and non-digital orthopantomograms (OPGs).

**Fig. 1:**
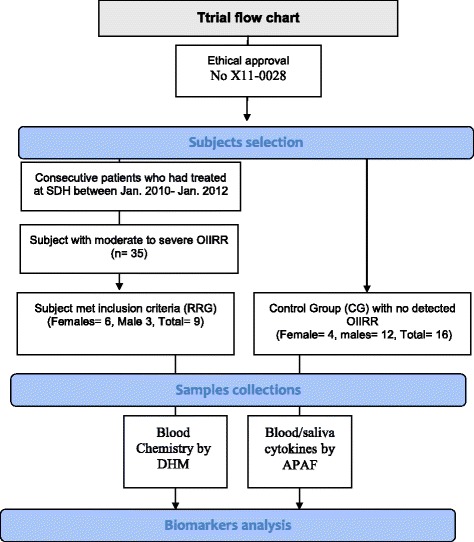
Study flow chart

The 35 identified participants were re-evaluated against specific inclusion and exclusion criteria. The inclusion criteria involved participants who were (a) willing to participate in the research; (b) finished their orthodontic treatment within 2 years and not receiving any active orthodontic treatment at the time of study and (c) the duration of their previous active orthodontic treatment should be less than 24. Participants with (a) active systemic disease; (b) history of allergy; (c) history of dental injury including but not limited to endodontic, periodontal, and physical; (d) morphological variation of resorbed teeth at the start of treatment; and (e) recent oral infections within 1 month such as flu, colds, herpes zoster and other oral ulcerations were excluded. The justification for this strict criteria was based on the fact that literature had shown that oral infection and dental trauma, and orthodontic treatment at the time of sample collection, could alter cytokine expression and induce root resorption not only to the individual tooth but surrounding teeth [3]. Following the application of these criteria, 25 participants had been excluded. Henceforth, only nine participants (mean age of 23 + 2.9 years) remained, namely root resorption group (RRG). Three subjects of the RRG had moderate OIIRR and the remaining had severe OIIRR, the RRG consisted of six females (mean age 22+ 2.2 years) and three males (mean age 25+ 3.6 years). Data from 16 participants, who met the previously mentioned inclusion/exclusion criteria and had not developed significant OIIRR, were used as a control (CG) (Fig. 1).

### Assessment of OIIRR

**Fig. 2:**
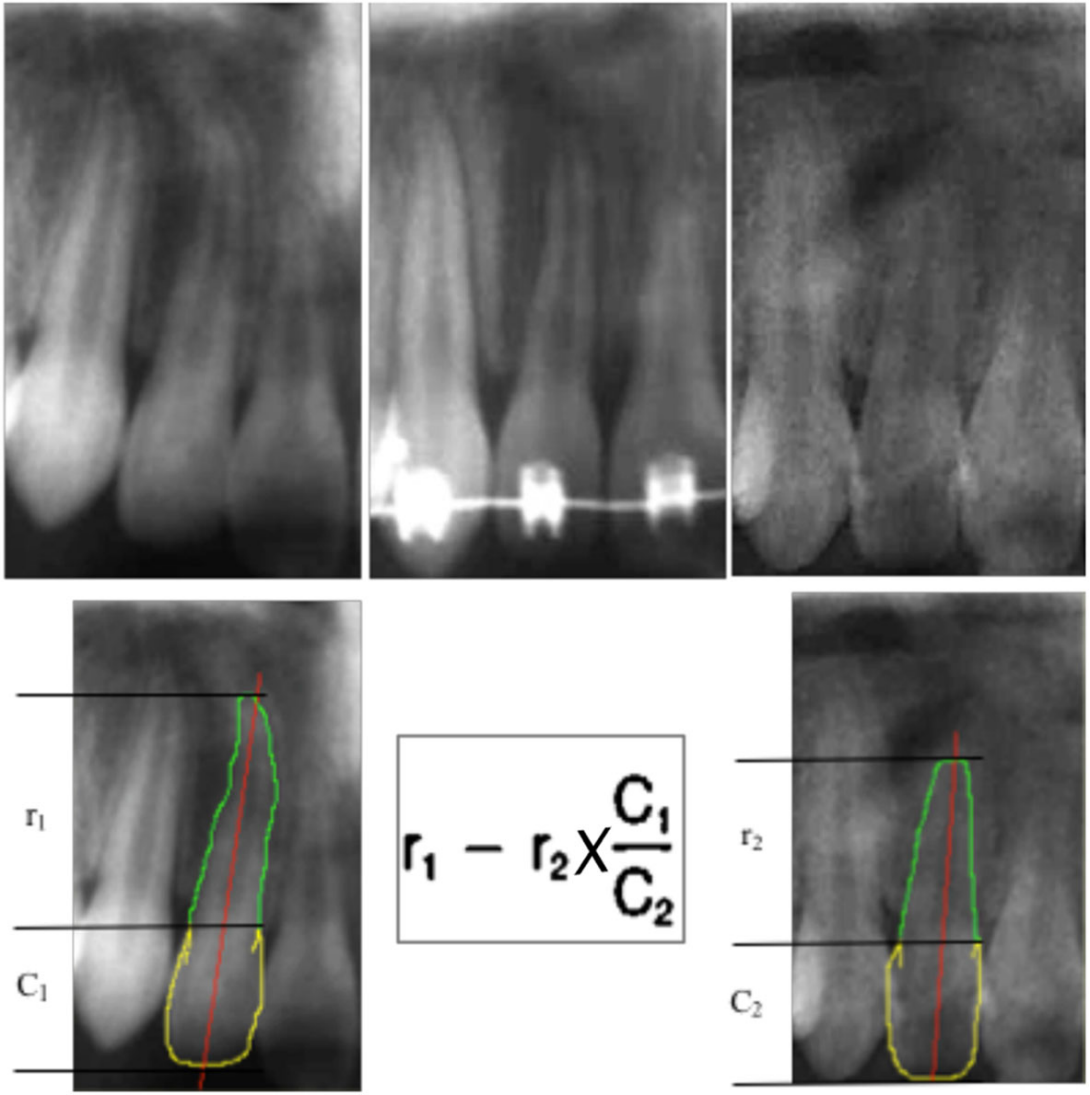
Cropped OPG of a participant  exhibiting moderate OIIRR of tooth No. 12 with equation used to assess the degree of OIIRR

Although a periapical radiograph is considered the gold standard for assessment of OIIRR, as this study was retrospective in nature, OPGs were available and hence used in this study. The degree of OIIRR was assessed from the OPG using the equation (Fig. 2) *as proposed by Linge and Linge  in 1991* [30]:

$$ OIIRR = \left(r 1\mathit{\hbox{-}}r 2\right)*\left(C 1/C 2\right) $$

Magnification error (C1/C2) developed using the ratio of the pre- (C1) and post-treatment (C2) radiographical crown height. The non-adjusted degree of OIIRR (r1-r2) was calculated as the difference between pre-treatment (r1) and post-treatment (r2) radiographical root length.

### Blood and saliva sample collection and analysis

Fasting for 12 h before blood and saliva collection had been requested from participants. Blood chemistry had been assessed through the collection of 33 mL of fasting blood by a phlebotomist at Douglass Hanly Moir Pathology (DHM) in St Vincent’s Clinic. Participants were also asked to brush their teeth in the clinic without toothpaste and to rinse with de-ionised water prior to collection of 10 mL of unstimulated saliva by expectoration into polypropylene tubes with protease inhibitors.

Blood and saliva samples were stored in a −80 °C freezer until transported to Australian Proteome Analysis Facility (APAF) at Macquarie University for analysis. Multiplex enzyme-linked immunosorbent assays (ELISA) was used to screen the samples’ saliva for cytokines, chemokines and several key enzymes that may play a role in OIIRR. Blood chemistry results were obtained from DHM pathology and analysed at APAF for significance. Table 1 shows blood chemistry factors and blood/saliva cytokines that were analysed by DHM and APAF individually.

**Table 1:** Biomarkers tested in patients with moderate to severe OIIRR

Blood chemistry	Blood and saliva—human high sensitivity cytokines/chemokines
Alkaline phosphate (ALP)	Granulocyte macrophage colony-stimulating factor (GM-CSF)
Calcitonin	Interferon-gamma (IFN-γ)
Calcium	Interleukin 1-beta (IL-1β)
Erythrocyte sedimentation rate (ESR)	Interleukin-10 (IL-10)
Full blood count (FBC)	Interleukin-12p70 (IL-12 p70)
IgE	Interleukin-13 (IL-13)
Osteocalcin	Interleukin-2 (IL-2)
Parathyroid hormone (PTH)	Interleukin-4 (IL-4)
Procollagen type I N-terminal peptide (P1NP)	Interleukin-5 (IL-5)
T-lymphocytes	Interleukin-6 (IL-6)
Thyroxine (Free T4)	Interleukin-7 (IL-7)
Vitamin 1,25-D (Vit-D)	Interleukin-8 (IL-8)
	Tumour necrosis factor-alpha (TNF-α)

### Statistical analysis

#### Cytokine expression analysis

Fluorescence intensity (FI) was used as the outcome measure for the concentration of cytokine expression in CG and RRG. Boxplots plotting FI versus cytokine expression for each diagnosis group allowed for a visual indication of data distribution and trends. A two-way analysis of variation (ANOVA) was conducted to investigate the effect of diagnosis on fluorescence through the use of the following mixed model calculation:

$$ \boldsymbol{Log}\left(\boldsymbol{F}\boldsymbol{1}\right)\sim \boldsymbol{Diagnosis}*\boldsymbol{Cytokine}+\boldsymbol{Sample}+\boldsymbol{Gender}+\left(\boldsymbol{1}\left|\boldsymbol{Age}\right.\right)+\left(\boldsymbol{1}\left|\boldsymbol{Patient}\right.\right)+\left(\boldsymbol{1}\left|\boldsymbol{Generalised}\;\boldsymbol{Ethnicity}\right.\right) $$

This accounts for the random effects of patients, age and ethnicity, and the fixed effects of diagnosis, sample, gender and cytokines. Besides, a one-way ANOVA was conducted to investigate the effect of diagnosis on blood and saliva cytokine. All statistical analysis was conducted using R Statistical Software (Foundation for Statistical Computing, Vienna, Austria) and the statistical significance was set at the *p* < 0.05 level.

#### Blood chemistry analysis

Chi-square tests were conducted to clarify the association of blood proteins’ levels and diagnosis. Both CG and RRG groups had been subclassified into three categories: increased, decreased and no difference group, according to the level of the significantly changed blood proteins.

## Results

### Cytokine expression

**Fig. 3:**
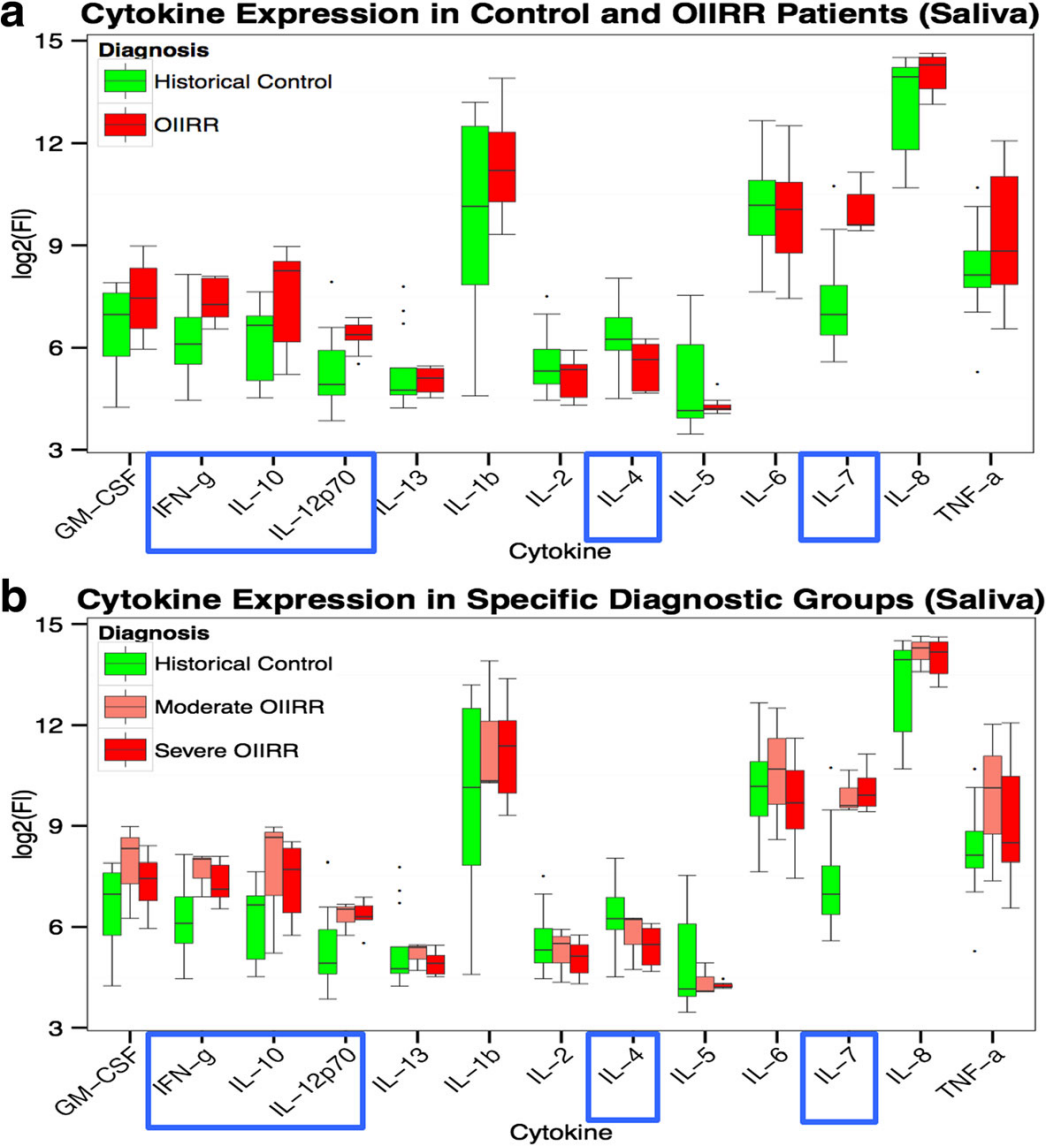
Cytokine expressions in saliva samples according to (**a**) general diagnosis of OIIRR comprising of severe and moderate root resorption groups compared to CG and (**b**) specific diagnosis of moderate and severe OIIRR compared to CG. Cytokines that showed significant changes in their expression are identified by *blue boxes*

Cytokine expression, as expressed by FI values, revealed significant findings in saliva samples of RRG patients. There was a statistically significant decrease in cytokine expression of IL-4 (p = 0.05) and a significant increase in cytokine expression of IFN-γ (*p* = 0.01), IL-10 (*p* = 0.03), IL-12p70 (*p* = 0.02), and IL-7 (*p* = 0.0001) in RRG compared to CG (Fig. 3a). However, there was no significant difference between moderate and severe RRG itself (Fig. 3b).

**Fig. 4:**
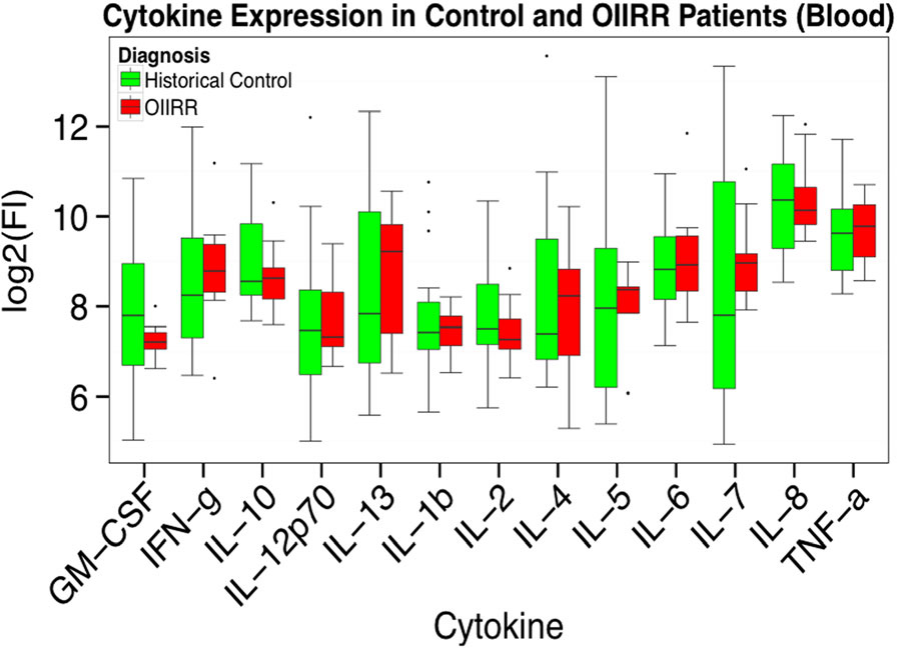
Showed that cytokine expression from blood samples according to RRG versus CG

Figure 4 showed that cytokine expression from blood samples according to RRG versus CG revealed no significant difference (*p* > 0.05). Likewise, there was no significant difference in cytokine expression between male and female subjects (*p* > 0.05).

### Blood chemistry

Blood chemistry results showed normal level for all proteins factors in both CG and RRG, except for osteocalcin (*p* = 0.039) and procollagen Type I N-Terminal Peptide (P1NP) (*p* = 0.033) concentrations. The proportion of participant who showed an increased osteocalcin concentration was almost six times more in CG (62.5%) than RRG (11.1%). The proportion of participants who presented with an elevated P1NP concentration was higher in CG (75%) than those in RRG (22.2%). None of the participants in both groups had experienced a drop in P1NP and osteocalcin concentration. Furthermore, blood chemistry’s trend tended to fit more closely with gender than with diagnosis; osteocalcin and P1NP levels were significantly elevated in male’s subgroup of the controlled sample compared to the counterpart (*p* < 0.05). On the other hand, there was no significant gender difference in blood factors among moderate and severe RRG (*p* > 0.05). However, subdividing the main groups by their gender further reduced sample numbers, and hence findings should be evaluated with caution.

## Discussion

This pilot study identified that cytokine expression between different diagnostic groups is more sensitive using saliva than in blood samples. In comparison to gingival crevicular fluid (GCF), saliva as a source to identify cytokines is considered remote from the root inflammatory zone, which means least overlapping of cytokines expressions [31, 32]. Thus, sampling saliva, as a chair-side screening test, is both affordable and easy compared to collecting blood or GCF. However, this study also showed that taking saliva samples might provide some indication of systemic reaction to potential inflammation.

The concept that pro-inflammatory cytokines are resorptive factors and anti-inflammatory cytokines are anti-resorptive factors is an oversimplified view and one that is changing. In our study, although the significant increase in IL-7 and a decrease in IL-4 could be linked specifically to root inflammatory phase, the increase in anti-inflammatory cytokines IL-10, IL-12p70 and IFN-γ was also significant. The anti-inflammatory cytokines may be upregulated to stimulate a constant state of remodelling in the bone akin to the body’s defence mechanism. Cytokines mediate bone damage by driving the differentiation and activation of the bone-resorbing cell, the osteoclast [33], as well as playing an essential role in immune cell development and immune-regulation. More recent evidence suggested that the effect of cytokine levels’ alterations is not a singular effect, that is, cytokines that promote inflammation can also have anti-inflammatory and immunosuppressive actions [34]. O’Shea et al. suggested that the combination of cytokines exerts variable effects at different times during autoimmune disease processes. IFN-γ, for example, has been shown to have multiple roles in autoimmunity; one as a mediator in autoimmune disease and another as having a protective role [34]. Thus, based on the findings of our study, it was difficult to precisely define the link between cytokines in OIIRR based on the small sample size.

A lack of significant difference in cytokine expression between genders is similar to the findings of Linge and Linge [3]. They noted that there was no connection between sex and severity of root resorption; therefore, if elevated levels of certain cytokines are linked to root resorption, gender may not play a role in affecting cytokine expression.

Osteocalcin and P1NP seemed to be the only blood factors that showed a significant difference between CG and RRG. However, gender appeared to have a more significant effect on osteocalcin and P1NP levels than diagnosis. It is important to isolate the effects of age and gender on blood osteocalcin and P1NP levels since bone turnover can be upregulated as a natural process of human growth. Previous research has shown that the distribution of osteocalcin at different sites of remodelling may be age- and gender-related changes [35]. Another study has revealed pubertal increases in P1NP level in both sexes [36]. Therefore, blood osteocalcin and P1NP may not be useful clinical markers to predict the susceptibility of patients to OIIRR as often these patients are receiving treatment at stages of natural growth and development.

This study applied computations to estimate radiographical magnification differences (RMD) between pre- and post-treatment OPGs taken by different operators and machinery [30]. Nevertheless, it was limited in its capacity to account for tilting of teeth especially maxillary and mandibular anterior teeth which are the most commonly affected teeth with extensive OIIRR [4, 37]. Another factor that should be considered in this study is the radiographical judgemental errors (RGE) in equating roots’ length from the OPGs. RGE is the result of film-related factors and/or radiographical conspicuity. Film-related factors include variation in radiographical brightness and contrast between pre- and post-treatment radiographs [38]. While conspicuity is a radiographic term which describes the background ‘noise’ caused by adjacent anatomical structures causing difficulty in assessing the examination area [8, 38], conspicuity arose when root apices were outside the narrow focal trough [39], incompletely developed roots or when the cervical vertebrae and other anatomical structures obscured the root apices. To overcome RGE and IGE, digital subtraction radiography (DSR) is recommended in prospective researches. DSR is a technique that requires the subtraction of one radiographic image from another, provided these two images have identical and reproducible projections of the same anatomic region [8, 9, 38]. For reproducibility purpose of DSR, bite registration with polyvinylsiloxane silicon impression material, invested into the bite block of a beam-guiding device, is used [8, 38]. After a series of radiographs are obtained at different time intervals, stable horizontal and vertical landmarks can be selected and eliminated in order to ensure measurement accuracy of the periapical regions between radiographs [38].

It is worth mentioning that many eligible participants deterred from consenting to participate in this pilot study and a repeated measurement of the saliva and blood sample were not undertaken to decrease the risk of bias. The main reason was that this study required large volumes of saliva and blood sample to be sent for laboratory testing. Recently, the use of microfluidic biosensors (MFB) as part of point-of-care diagnostic portable devices (PoC) was recommended in the monitoring of cytokine expression in a clinical setting [40]. The detection times of MFB are relatively faster than conventional laboratory testing, taking only from a few seconds to tens of seconds to process the sample. Another advantage of MFB is the small sample volume that could be used, ranging from micro-litres to nano-litres [41]. Prospect adoption of PoC devices in orthodontic practices could be used for accurate detection of cytokine expression and level changes. Thus, it would be possible to ‘red-flag’ patients that are more at risk of extensive OIIRR earlier. Subsequently, patients at risk can receive adequate modification in their orthodontic treatment, perhaps even pausing treatment which would allow the regenerative properties of cementum to repair the root surface, thereby minimising irreversible loss of root structure [6, 7].

Finally, as the relationship between cytokines and OIIRR in our study was based on the small sample size, future prospective studies with larger samples may yield interesting results, in particular the pre- and post-orthodontic level of cytokine.

## Conclusions

This study found that saliva might be more efficient in measuring changes in cytokine expression than blood. Osteocalcin and P1NP appeared to be the only blood factors that showed a significant difference between groups.

The weak association between treatment variables and patient characteristics with the degree of root resorption suggests that increased expression of pro-inflammatory, as well as anti-inflammatory cytokines, may be determinants in the development of moderate to severe OIIRR. However, the expression of cytokines may also be affected by any other potential inflammation in the body.

Further investigations could utilise digital subtraction radiography techniques and microfluidic biosensors, in a larger group of patients, to provide a more accurate and detailed insight into the mechanisms of OIIRR. Cytokine expression at different time intervals, during and after orthodontic treatment, may also give more information about the inflammatory nature of OIIRR.
